# Abdominal compartment syndrome – the prevention and treatment of possible lethal complications following hip arthroscopy: a case report

**DOI:** 10.1186/1752-1947-8-368

**Published:** 2014-11-14

**Authors:** Kinga Ciemniewska-Gorzela, Tomasz Piontek, Andrzej Szulc

**Affiliations:** 1 Rehasport Clinic Poznan, Clinic of Pediatric Orthopedic Surgery UM in Poznan, Rehasport Clinic, ul. Górecka 30, 60-201 Poznań, Poland; 2 Clinic of Pediatric Orthopedic Surgery UM in Poznan, Clinic of Pediatric Orthopedic Surgery, ul 28 czerwca 1956 r. 134/145, 61-495 Poznań, Poland

**Keywords:** Abdominal compartment syndrome, Complication, Hip arthroscopy

## Abstract

**Introduction:**

Intra-abdominal hypertension and abdominal compartment syndrome have been increasingly recognized as a hip arthroscopy complication over the past decade. In the absence of consensus definitions and treatment guidelines, the diagnosis and management of intra-abdominal hypertension and abdominal compartment syndrome remains variable from institution to institution.

**Case presentation:**

We report the occurrence of the extravasation of fluid into the abdomen during arthroscopic treatment of femoroacetabular impingement combined with resection of trochanteric bursa and our management of the condition in a 55-year old Caucasian woman.

**Conclusions:**

We present an algorithm of treatment of abdominal compartment syndrome, as a hip arthroscopy complication, according to the consensus definitions and recommendations of the World Society of the Abdominal Compartment Syndrome. In the algorithm options, we have included paracentesis and percutaneous catheter decompression as the main point of treatment. Our algorithm will have a broader clinical impact on orthopedic surgery, anesthesiology and emergency medicine.

## Introduction

By having an upgraded understanding of hip anatomy, arthroscopy has been established as a standard technique in hip surgery. The potential complications of hip arthroscopy are: neurovascular traction injury, direct trauma to neurovascular structures, compression injury to the perineum, traction fixation device injury, arthroscopy-related trauma, breakage of instruments, vascular insult to the femoral head, infection, and fluid extravasations [1,2]. The development of the hemodynamically compromising abdominal compartment syndrome (ACS) after hip arthroscopy is a rare but possible and lethal complication [1-8]. Early signs of abdominal pain due to the accumulation of fluid in the abdominal cavity may be masked by regional anesthesia and sedation [5]. Pathophysiologically, it deranges cardiovascular hemodynamics, respiratory and renal functions, and may eventually lead to multi-organ failure [9-12]. Significant progress has been made over the past decade towards understanding the etymology and pathophysiology surrounding intra-abdominal hypertension (IAH) and ACS. The absence of consensus definitions and treatment guidelines, however, has led to confusion over both the prevalence of IAH and ACS, as well as the most effective treatment strategies for such patients. Failure to recognize and appropriately treat ACS is uniformly fatal, whereas, prevention and/or timely intervention has been associated with marked improvements in organ function, and overall patient survival [9-11]. The survey experts in the field of hip arthroscopy from the Multicenter Arthroscopy of the Hip Outcomes Research Network (MAHORN) Group had determined the frequency of symptomatic intra-abdominal fluid extravasation (IAFE) after arthroscopic hip procedures. Potential risk factors had been identified among 40 cases of symptomatic IAFE [13]. The main disadvantage of the study had been the fact that the recommended strategies of treatment were based on the experience of experts in the field of hip arthroscopy and not in the field of the World Society of the Abdominal Compartment Syndrome (WSACS). Moreover, the study had not presented a clear algorithm of treatment, which is very important to avoid mistakes in emergency treatment.

The presented case highlights the need for surgeons to be aware of this rare but serious complication, and we indicate the steps necessary to manage it promptly.

In this manuscript we aim to report a life-threatening case of primary acute ACS resulting from iatrogenic extravasation after hip arthroscopy. We also reviewed the literature and created an algorithm of treatment of ACS according to the WSACS, and additionally reviewed consensus definitions and recommendations.

## Case presentation

A 55-year-old Caucasian woman (53kg, 160cm; body mass index 20.7kg/m^2^) had been scheduled for right hip arthroscopy, partial labral resection for a labral tear, psoas release of her right hip, and resection of trochanteric bursa in relation to the gluteus medius tendon. She consented to undergo spinal anesthesia and pharmacological sedation with propofol in constant 10 to 15mL/hour infusion for this procedure. Standard monitoring (electrocardiogram), noninvasive blood pressure monitoring and pulse oximetry were used. Following uneventful spinal anesthesia, at the fourth lumbar interspace using 3mL isobaric bupivacaine 0.5%, she was positioned supine, prepped, and draped. Supplemental oxygen had been provided by a nasal cannula. An infusion pump with propofol had been admitted. Traction had been applied to the lower extremities and was confirmed by a fluoroscopic examination. The anterior and anterolateral arthroscopic portals were used as standard approaches to the right hip. The arthroscope and arthroscopic instruments were introduced into the joint under fluoroscopic guidance, and 0.9% sodium chloride. Interportal capsulotomy had been performed to facilitate instrument navigation and arthroscopic visualization. After hip diagnostic arthroscopy the damaged labrum had been debrided back to stable margins. The acetabular rim had been adjusted with an arthroscopic bar. The psoas tendon had been released at the level of the capsule via a transcapsular approach. The femoroplasty for the decompression of the small cam deformity had been performed.

The DYONICS™ 25 Fluid Management System was used. This high-flow pump pumps fluid with a maximum flow rate of 1.5L/minute for procedural speed and efficiency. A total of eight 3L bags were used for the procedure. The procedure lasted approximately 2 hours, and anesthetic management was uneventful until the final 5 minutes of surgery. To ensure no increases in abdominal tension, we had performed a preoperative abdominal routine examination under anesthesia before prepping and draping, in order to obtain a baseline followed by a series of abdominal examinations throughout the procedure. The remaining 10 minutes of surgery had been devoted to work outside of the joint in paratrochanteric bursa and gluteus medius origin. At the final stage of surgery she became acutely short of breath with a tachycardia above 100. Acute abdominal pain was visible. The upper body warming blanket had been removed, revealing significant abdominal distension. Systolic blood pressure was low at approximately 90mmHg. The surgeon was notified and surgical closure procedures swiftly completed the operation. The incisions were closed, and preparations were made to transfer her to the recovery room. She remained hemodynamically stable for the rest of the procedure. Although the cause of abdominal distension was not known at that point, there was an assumption that it had resulted from either large amounts of blood or arthroscopy irrigation fluid, or both, in her abdomen. A general surgeon was consulted; the surgeon recommended ultrasonographic assessment of her abdomen for an evaluation of the type and quantity of fluid. The ultrasonographic examination was performed and clear fluid was found. Right lower quadrant paracentesis was performed and 3L of the clear, colorless fluid was drained from her abdomen, showing an immediate improvement in her hemodynamic status.

### The technique of paracentesis

She was positioned in a semi-sitting position tilt, on her right side. Her abdomen was sterile. Right lower quadrant palpation had been performed before the peritoneal fluid removal. The sterile technique of the paracentesis was done using a 14G catheter. The catheter had been carefully percutaneously inserted into the abdominal cavity and left *in situ* for slow drainage.

## Discussion

According to WSACS definitions, primary ACS (formerly termed as surgical, postoperative, or abdominal ACS) is characterized by the presence of acute, or subacute IAH, of relatively brief duration [9,10]. Such duration occurs as a result of an intra-abdominal reason such as: abdominal trauma, ruptured abdominal aortic aneurysm, hemoperitoneum, acute pancreatitis, secondary peritonitis, retroperitoneal hemorrhage, or liver transplantation. Fluid extravasations after hip arthroscopy also can lead to ACS [9,10]. This potentially lethal complication of hip arthroscopy highlights the need for orthopedists to be aware of this rare but serious complication, and of the steps needed to be undertaken in order to manage it promptly. Although Fowler and Owens [6] mention death after hip arthroscopy among Bartlett’s patients, Bartlett *et al.*[3] do not confirm such information in their original paper. The prevalence and etiological factors for IAH and ACS after hip arthroscopy are being reviewed.

**Figure 1:**
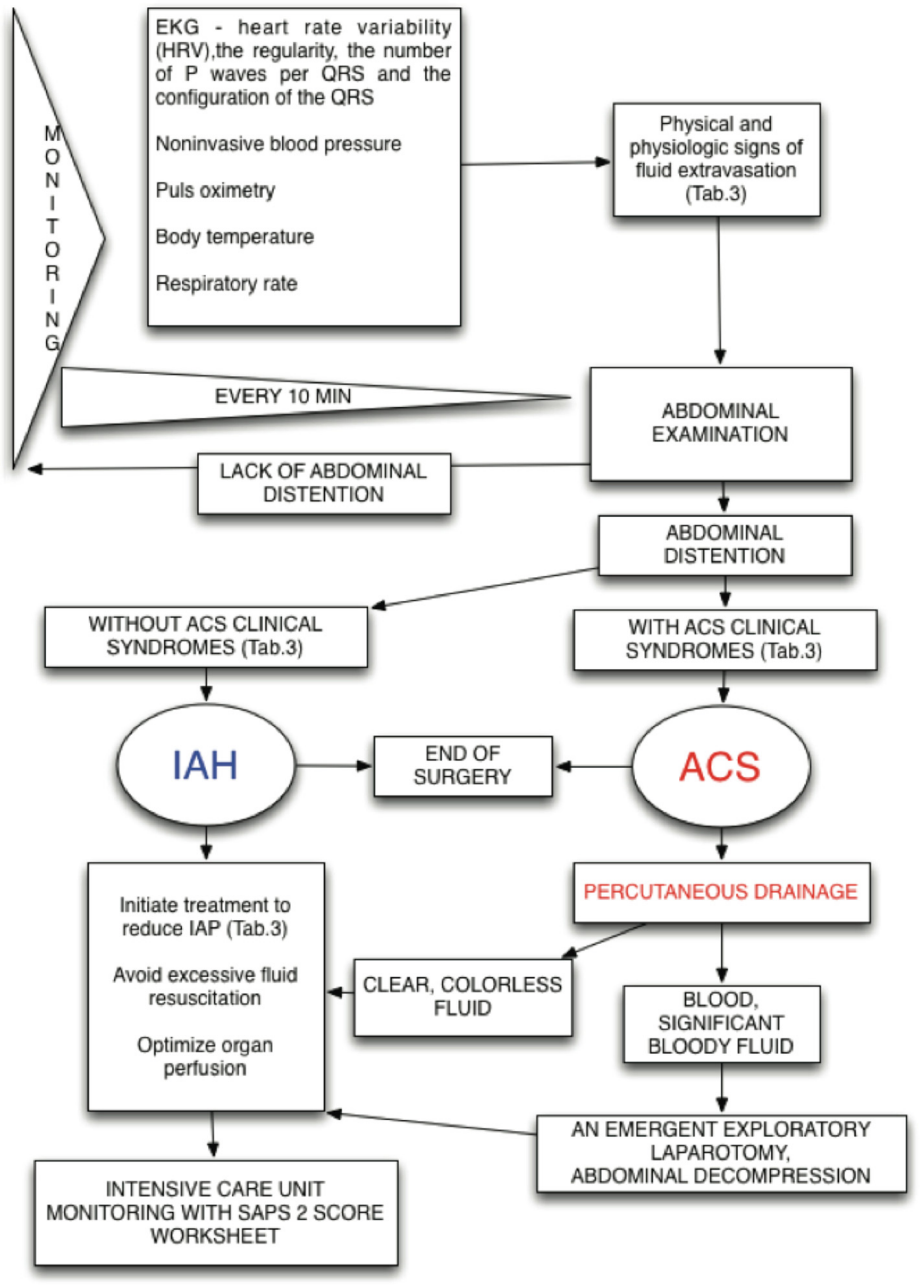
Intra-abdominal hypertension/intra-abdominal compartment syndrome management algorithm.

Evidence-based medicine treatment guidelines have been presented to facilitate the diagnosis and management of IAH and ACS (Figure 1). The choice (and success) of the medical management strategies given in Figure 1 is strongly related to both the etymology of the patient’s IAH/ACS, and the patient’s clinical situation. The appropriateness of each intervention should always be considered prior to implementing these interventions in any individual patient. This paper presents the case of ACS after hip arthroscopy, treated successfully with paracentesis and percutaneous catheter decompression. The pathophysiology of IAH and ACS in hip arthroscopy has been described in many papers [3-8]. The details have been collected in Tables 1 and 2. Many authors have suggested the only possible way for the irrigation fluid to flow is by a retroperitoneal course along the iliopsoas muscle and the iliac vessels with intraperitoneal perforation along their course [4-8]. The proposed mechanism of extravasation is similar to our case. After having performed an analysis of all the cases described in the literature, and having collected data from our case, we were unable to indicate any clear risk factors of IAH and ACS in hip arthroscopy. In contrast to other authors’ suggestions, a prolonged surgical time (95 – 165min in analyzed cases), and patient’s position (four cases in supine positions and three cases in lateral positions) or specific surgical procedures such as extra-articular surgery (many types of surgical procedures), we could define no clear risk factors of IAH or ACS. We agree with Verma and Sekiya [7], and hypothesize that our patient had had communication between the retroperitoneal and peritoneal cavity, which allowed for this amount of fluid extravasation. This hypothesis is also supported by nephrologists’ experience with acute ultrafiltration failure (AUFF), which is an important clinical problem among patients having peritoneal dialysis (PD). Lam *et al.* reported the cases of three patients on continuous ambulatory PD who developed reversible ultrafiltration failure secondary to retroperitoneal leakage (RPL) [14]. Lam *et al.* also reported that during the 5-year study period, 36 patients in a cohort of 743 patients on maintenance PD developed AUFF. Of these 36 patients, 23 were found to have RPL, which was confirmed by either computed tomography (n=16) or magnetic resonance imaging (n=7) [15]. The authors concluded that RPL was not uncommon among patients with AUFF. There is a high possibility for the occurrence of RPL among patients with AUFF and additionally having a history of hernia or pleuroperitoneal communication. In addition, the commonly used high-flow pump for hip arthroscopy can pump fluid with a maximum flow rate of 1.5L/minute; the catheter for PD can transmit fluid with a maximum flow of 0.5L/minute and would fill up the abdomen with 2L of fluid in 5 to 10 minutes. This means that even 2 to 3 minutes of surgery without abdomen examinations may be sufficient to overlook dangerous abdominal extravasation.

**Table 1:** Cases of fluid extravasation after hip arthroscopy – demographic data and hip operative procedure

**Authors, year of publication and reference number**	**Age and gender**	**Hip treatment procedures**	**Time of surgery (minutes) and surgical position**
Bartlett *et al*., 1998 [3]	50-year-old man	1. Loose-body removal 13 days after acetabular fracture of both columns treated with open reduction–internal fixation using an ilioinguinal approach	135/lateral position
Haupt *et al.*, 2008 [4]	15-year-old girl	1. Capsulotomy	105/lateral position
		2. Adhesion releases after open acetabular retroversion corrected by trimming the anterosuperior rim with reattachment of the labrum.	
Sharma *et al.*, 2009 [5]	45-year-old woman	1. Limited capsulectomy	160/supine position
		2. Labral repair	
		3. Psoas release	
Fowler and Owens, 2010 [6]	42-year-old man	1. Limited capsulectomy	95/lateral position
		2. Psoas tenotomy	
		3. Debridement of the anterior and superior labrum and pincer-type lesion	
Verma and Sekiya, 2010 [7]	21-year-old woman	1. Capsulotomy	139/supine position
		2. Iliopsoas tenotomy	
		3. Osteoplasty to treat the femoroacetabular cam impingement	
Ladner *et al.*, 2010 [8]	42-year-old woman	1. Limited capsulectomy	165/supine position
		2. Debridement of the irreparable large labral tear	
		3. Chondroplasty on the acetabular rim	
		4. Osteoplasty of the femoral head-neck junction and acetabular rim	
Current case	55-year-old woman	1. Capsulotomy	120/supine position
		2. Iliopsoas tenotomy	
		3. Osteoplasty to treat the femoroacetabular pincer and cam impingement	
		4. Resection of trochanteric bursa in relation to gluteus medius tendon	

**Table 2:** **Cases of fluid extravasation after hip arthroscopy – intra-abdominal hypertension**/**abdominal compartment syndrome treatment and outcomes**

**Author and Reference number**	**Intra-abdominal hypertension/acute abdominal compartment syndrome clinical signs**		**Intra-abdominal hypertension/acute abdominal compartment syndrome treatment procedures**	**Treatment results**
	**Abdomen**	**Others**		
Bartlett *et al.*[3]	Significant abdominal distension	Cardiopulmonary arrest	1. Nonoperative medical management	Despite prolonged asystole, the patient survived without neurologic sequelae
			2. An emergent exploratory laparotomy closed primarily	
Haupt *et al.*[4]	Diffuse abdominal pain 4 hours after surgery	1. Body temperature decreased from 36.3° to 34.5°C at the end of the operation	Nonoperative medical management	The irrigation solution was absorbed the next day
		2. Concurrent neurologic symptoms, resembling absence seizures occurred		The neurologic symptoms disappeared without treatment
Sharma *et al.*[5]	Significant abdominal distension	1. Acute hypotensive with a systolic blood pressure of 60–70mmHg	1. Nonoperative medical management	Immediate improvement in the patient’s hemodynamic status
		2. Unresponsive	2. Urgent mini-laparotomy and then diagnostic laparotomy	
		3. Apnoeic		
		4. Lower extremities appeared cyanotic no pulse could be palpated in either leg		
Fowler and Owens [6]	Abdomen extremely distended	1. Elevated bladder pressures (42mmHg)	1. Nonoperative medical management	Asymptomatic in his right hip and groin but is continuing follow-up by a general surgeon for abdominal complaints related to his incision and abdominal compartment syndrome
		2. An increased peak inspiratory pressure, thus preventing extubation	2. An emergent exploratory laparotomy. The abdomen was left open, and a wound vacuum was placed.	
Verma and Sekiya [7]	Distended and firm abdomen but easily compressible	1. Hypothermia during the surgical procedure	Nonoperative medical management	The irrigation solution was absorbed
		2. Right labia was asymmetrically enlarged		
Ladner *et al.*[8]	Abdomen noticeably distended	1. Core body temperature remained above 36.8°C.	Paracentesis – no fluid was obtained. A computed tomography scan after paracentesis showed a copious amount of fluid in the intraperitoneal area and a small amount in the retroperitoneal area	The irrigation solution was absorbed the next day
		2. At no time was her respiratory or cardiac function compromised based on clinical examination, blood pressure, heart rate, arterial blood gas values, and electrocardiographic data		
Current case	Abdomen extremely distended	1. Acute hypotension with a systolic blood pressure of 60–70mmHg	Paracentesis and percutaneous slow drainage	Immediate improvement in the patient’s hemodynamic status
	Abdominal pain	2. Unresponsiveness		
		3. Shortness of breath		

In our opinion, hip arthroscopy per se, places the patient at high risk for primary ACS. Intra-abdominal fluid extravasation is a known complication (0.16% incidence) of hip arthroscopy which needs to be informed to the patient prior to the procedure.

The indication of risk factors of IAH and ACS in hip arthroscopy needs further research on a larger scale of cases. It appears that patients with a history of hernia or pleuroperitoneal communication are more likely to be classified as at higher risk of ACS before hip arthroscopy. However, complications are very rare, and because of that we recommend to collect all cases in a WSACS database, or precisely describe the cases in medical papers. This should allow us to collect a larger amount of data for further analysis. The primary mission of WSACS is to promote research, foster education, and improve the survival rate of patients with IAH and ACS, by sharing information on effective management strategies [9,10]. The WSACS provides instructional materials (IAH and ACS consensus definitions and clinical practice guidelines) to upgrade the education of physicians, nurses, respiratory therapists, and other healthcare providers [16]. WSACS has recently published two consensus documents detailing the current state-of-the-art diagnosis and management of IAH/ACS [9,10]. No single management strategy can be uniformly applied to a patient with IAH/ACS. Several fundamental management concepts, however, remain appropriate across all cases. While surgical decompression is commonly considered the only treatment for IAH/ACS, nonoperative medical management strategies are now recognized as playing a vital role in both the prevention and treatment of organ dysfunction and failure due to elevated intra-abdominal pressure (IAP) [11,12]. Appropriate treatment and management of IAH and/or ACS is based upon four general principles: (a) serial monitoring, (b) optimization of systemic perfusion and a patient’s organ function with elevated IAP, (c) institution of specific medical procedures (with paracentesis and percutaneous catheter decompression as a main point of treatment) to reduce IAP and the end-organ consequences of IAH/ACS, and (d) prompt surgical decompression for refractory IAH [9,10]. An algorithm for the management of a patient with IAH/ACS has been proposed in Table 3.

**Table 3:** Algorithm-related physical and physiological signs of fluid extravasation syndrome and abdominal compartment syndrome: initiated treatment options to reduce intra-abdominal pressure

**Physical and physiologic signs of fluid extravasation**	**Abdominal compartment syndrome clinical syndromes**	**Initiate treatment to reduce intra-abdominal pressure**
Cardiac arrhythmias	Systolic blood pressure less than 90mmHg or need for catecholamine support	Improve abdominal wall compliance
		Sedation and analgesia. Neuromuscular blockade. Avoid head of bed >30 degrees
Hypotension	PaO_2_ 60mmHg or less or need for mechanical ventilation	Correct positive fluid balance
		Avoid excessive fluid resuscitation. Diuretics. Colloids/hypertonic fluids. Hemodialysis/ultrafiltration
Oxygen saturation <95	Need for hemodialysis or creatinine level greater than 177umol/L after rehydration	Organ support. Maintain APP >60mmHg with vasopressors. Optimize ventilation, alveolar recruitment. Use transmural airway pressures
		Pplattm = Pplat – IAP. Consider using volumetric preload indices. If using PAOP/CVP, use transmural pressures
		PAOPtm = PAOP – 0.5 × IAP CVPtm = CVP – 0.5 × IAP
Hypothermia (core temperature <35°C)	Unresponsiveness	Evacuate intraluminal contents. Nasogastric decompression. Rectal decompression. Gastro-/colo-prokinetic agents
Shortness of breath	Shortness of breath/apnea	
Peak pressure ↑ on artificial ventilation		

*Abbreviations*: APP abdominal perfusion pressure, *CVP* central venous pressure, *IAP* intra-abdominal pressure, *PaO*_2_ oxygen partial pressure arterial, *PAOP* pulmonary artery occlusion pressure, *Pplat* plateau pressure, *Pplattm* transmural plateau pressure, *tm* transmural.

Among patients having undergone trauma or elective surgery, operative decompression is advocated if noninvasive treatment options prove insufficient. Decompression laparotomy reduces IAP instantaneously and is often a life-saving procedure [9-12]. Given the morbidity of open abdominal decompression, less invasive means to reduce IAP would certainly be appealing. Percutaneous catheter decompression represents a less invasive method for treating IAH or primary ACS due to free intra-abdominal fluid. This technique appears to be effective in reducing IAP, and potentially corrects the IAH-induced organ dysfunction. Possible performance under ultrasound, or computed tomography guidance and percutaneous decompression among appropriate patients, appears to be effective in solving IAH/ACS and avoiding the need for surgical decompression [11,12]. In light of the potential benefits of avoiding abdominal decompression, we suggest that percutaneous catheter decompression should be considered among patients with intraperitoneal fluid or blood, who demonstrate symptomatic IAH or ACS.

## Conclusions

We have presented an algorithm of treatment of ACS, as a hip arthroscopy complication, according to the WSACS consensus definitions and recommendations. In the algorithm options we have included paracentesis and percutaneous catheter decompression as a main point of treatment. The authors anticipate that these definitions and recommendations will be dynamic and will change as new research is published. They will have a broader clinical impact across orthopedic surgery, anesthesiology and emergency medicine.

## Consent

Written informed consent has been obtained from the patient for publication of this case report and any accompanying images. A copy of the written consent is available for review by the Editor-in-Chief of this journal.

## Abbreviations

ACS: Abdominal compartment syndrome; AUFF: Acute ultrafiltration failure; IAFE: Intra-abdominal fluid extravasation; IAH: Intra-abdominal hypertension; IAP: Intra-abdominal pressure; MAHORN: Multicenter Arthroscopy of the Hip Outcomes Research Network Group; PD: Peritoneal dialysis; RPL: Retroperitoneal leakage; WSACS: World Society of the Abdominal Compartment Syndrome.

## Competing interests

The authors declare that they have no competing interests.

## Authors’ contributions

KC-G participated in the study design, sequence alignment, data interpretation and literature search, and drafted the manuscript. TP participated in study design, sequence alignment, data collection and interpretation, and coordination to draft the manuscript. ASz participated in the study design, data interpretation and helped to draft the manuscript. All authors have read and approved the final manuscript.
